# The Impact of N-terminal Acetylation of α-Synuclein on Phospholipid Membrane Binding and Fibril Structure*

**DOI:** 10.1074/jbc.M116.726612

**Published:** 2016-08-16

**Authors:** Aditya Iyer, Steven J. Roeters, Nathalie Schilderink, Bob Hommersom, Ron M. A. Heeren, Sander Woutersen, Mireille M. A. E. Claessens, Vinod Subramaniam

**Affiliations:** From the ‡ Nanoscale Biophysics Group, FOM Institute AMOLF, Amsterdam,; the § Nanobiophysics Group, MESA+ Institute for Nanotechnology, University of Twente, Enschede,; the ¶ Van't Hoff Institute for Molecular Sciences, University of Amsterdam, Amsterdam,; the ‖ BioImaging MS Group, FOM Institute AMOLF, Amsterdam, The Netherlands,; the ** M4I, The Maastricht MultiModal Molecular Imaging Institute, University of Maastricht, and; the ‡‡ Vrije Universiteit Amsterdam, De Boelelaan 1105, 1081 HV Amsterdam, The Netherlands

**Keywords:** amyloid, circular dichroism (CD), lipid-protein interaction, post-translational modification (PTM), protein conformation, protein self-assembly

## Abstract

Human α-synuclein (αS) has been shown to be N terminally acetylated in its physiological state. This modification is proposed to modulate the function and aggregation of αS into amyloid fibrils. Using bacterially expressed acetylated-αS (NTAc-αS) and endogenous αS (Endo-αS) from human erythrocytes, we show that N-terminal acetylation has little impact on αS binding to anionic membranes and thus likely not relevant for regulating membrane affinity. N-terminal acetylation does have an effect on αS aggregation, resulting in a narrower distribution of the aggregation lag times and rates. 2D-IR spectra show that acetylation changes the secondary structure of αS in fibrils. This difference may arise from the slightly higher helical propensity of acetylated-αS in solution leading to a more homogenous fibril population with different fibril structure than non-acetylated αS. We speculate that N-terminal acetylation imposes conformational restraints on N-terminal residues in αS, thus predisposing αS toward specific interactions with other binding partners or alternatively decrease nonspecific interactions.

## Introduction

α-Synuclein (αS)^5^ is an intrinsically disordered monomeric protein found in particularly high concentrations at the synaptic junctions of neuronal cells (1–3). Its physiological function and precise role in the etiology of Parkinson's disease remain, to date, unknown. The binding of αS to phospholipid membranes observed *in vitro* is thought to be relevant for its function in eukaryotic cells and may facilitate the αS aggregation cascade that possibly leads to neuronal cell death in Parkinson's disease. The phospholipid membrane binding and aggregation of αS have been extensively characterized *in vitro* (4–11). Although αS is known to be subject to post-translational modifications (2, 12), previous investigations used αS that was recombinantly expressed in bacteria, and are thus not post-translationally modified. Post-translational modifications (PTMs) such as phosphorylation, ubiquitination, or acetylation are used by eukaryotic cells to modulate protein conformation and/or function. More than 90% of eukaryotic cellular proteins are N terminally acetylated (13, 14) and it is now established that N-terminal acetylation is the predominant PTM in αS (15–17).

*In vivo*, the influence of N-terminal acetylation of αS on its aggregation into amyloid fibrils is unknown, whereas existing reports from *in vitro* experiments are contradictory (18–20). Considering the critical role of the N-terminal residues of αS in phospholipid membrane binding (12, 21, 22), N-terminal acetylation can reasonably be expected to affect, and perhaps even regulate, membrane binding. We therefore probed how this αS modification impacts the affinity of αS for phospholipid membranes and investigated how it affects the aggregation into amyloid fibrils. In this report, the membrane binding properties of bacterially expressed N terminally acetylated-αS (NTAc-αS) and αS purified from human erythrocytes (Endo-αS) was assessed by systematically varying charge density and cholesterol content of both large unilamellar vesicles (LUVs) and highly curved small unilamellar vesicles (SUVs) using circular dichroism (CD) spectroscopy. Our observations show that N-terminal acetylation does not significantly influence the membrane binding affinity of αS as a function of membrane anionic charge, cholesterol content, and curvature. The effect of acetylation is more pronounced in the kinetics of αS aggregation into amyloid fibrils. We used atomic force microscopy (AFM) and two-dimensional infrared spectroscopy (2D-IR) to extract qualitative and quantitative information on the structure of fibrils of NTAc-αS and Endo-αS (henceforth acetylated-αS) and WT-αS. Our results suggest that the fibril structure of both types of acetylated-αS is a well defined distribution of β-sheet structures differing markedly from WT-αS.

## Results and Discussion

**FIGURE 1.**
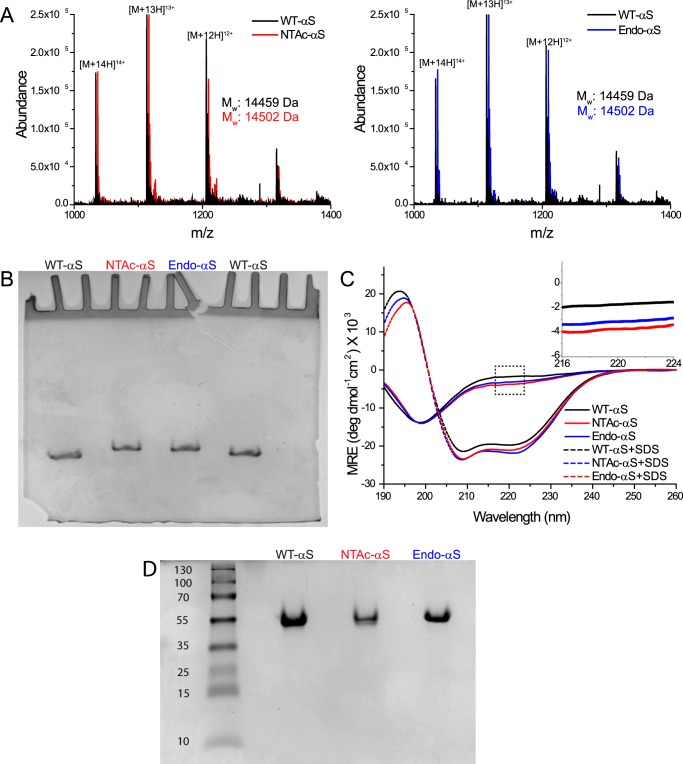
**Biochemical characterization of αS variants.** *A,* ESI-MS data of purified monomeric WT-αS, NTAc-αS, and Endo-αS, respectively. All samples were prepared in 10 mm ammonium acetate buffer with the concentration of αS monomers kept constant at 15 μm. For a given *m*/*z* value, the corresponding charge state is indicated. Molecular masses (*M*_r_) were calculated as follows: *m*/*z* value = [M + *x*H]^x+^. *M*_r_ = (*m*/*z* value × *x*) − *x. B,* acetic acid gel electrophoresis data of monomeric WT-αS, NTAc-αS, and Endo-αS. 5 μm of each protein sample was loaded into gels and as shown above, the relative migration of WT-αS was more than that of acetylated-αS, which migrated at similar positions. *C,* CD spectra showing the conformational transition from a random coil to a α-helix upon the addition of SDS micelles. The *inset* shows the slightly higher absorbance of acetylated-αS at 222 nm compared with WT-αS. All data obtained with WT-αS are depicted with *black*, with NTAc-αS with *red*, and Endo-αS with *blue* colors, respectively. *D,* native-PAGE gel of WT-αS, NTAC-αS, and Endo-αS showing absence of any higher ordered aggregates in either sample. A standard PageRuler^TM^ Plus pre-stained protein ladder was loaded in the *left-most lane* and the *numbers* correspond to molecular masses in kDa. A minute band appears in the WT-αS lane very close to the beginning of the resolving gel, which is larger than 250 kDa in size.

To confirm that the bacterially expressed NTAc-αS was correctly acetylated, we first characterized the purified WT-αS, NTAc-αS, and Endo-αS using electrospray ionization mass spectrometry (ESI-MS) and acetic acid gel electrophoresis. The ESI-MS results show that all three αS variants were monomeric with WT-αS having the expected molecular mass of 14,459 Da. The molecular mass of NTAc-αS (14,502 Da) confirms the presence of a single acetyl group in NTAc-αS as reported previously (23). The molecular weight of NTAc-αS was identical to that of Endo-αS suggesting the absence of any other post-translational modifications in Endo-αS (Fig. 1*A*). The slower migration of both acetylated-αS in the acetic acid gel electrophoresis experiment compared with WT-αS confirmed the loss of a positive charge upon αS acetylation (18); the gel also confirms the absence of any high molecular weight species (Fig. 1*B*) in our preparations (see also native PAGE gel in Fig. 1*D*).

To address the possible effect of N-terminal acetylation on the secondary structure of free and micelle-bound αS, we acquired CD spectra of both acetylated-αS and WT-αS in buffer with and without SDS micelles. The resulting spectra showed typical random coil and helix conformations for all three proteins in buffer solution and on SDS micelles, respectively (Fig. 1*C*). The slightly higher absorbance in the 222-nm region for the unstructured acetylated-αS (Fig. 1*C*, *inset*) agrees with the higher helical content of αS in solution observed in NMR experiments (18). These NMR experiments indicate that N-terminal acetylation impacts the first 12 residues in αS resulting in a small increase in the helical propensity (18). The stabilization of the α-helical structure in N terminally acetylated-αS is not unique but is generally observed in other proteins with this PTM (24, 25).

**FIGURE 2.**
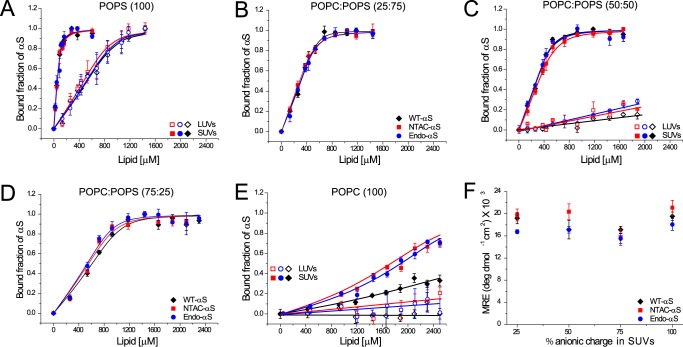
**Membrane binding characteristics of WT-αS, NTAc-αS, and Endo-αS.** All data obtained with WT-αS are depicted with *black diamonds*/*lines*, NTAc-αS with *red squares*/*lines,* and Endo-αS with *blue circles*/*lines. Open symbols* in *panels A, C*, and *E* represent data obtained in the presence of LUVs and *closed symbols* represent data obtained in presence of SUVs. Binding curves of αS to 100% POPS liposomes (*panel A*), 25:75 POPC:POPS (*panel B*), 50:50 POPC:POPS (*panel C*), and 75:25 POPC:POPS (*panel D*) showing no differences in membrane binding of acetylated-αS compared with WT-αS. Binding curves of αS to POPC liposomes show hardly any binding of αS to membranes of this composition but acetylated-αS has a slightly higher affinity for 100% POPC SUVs than WT-αS (*panel E*). Average MRE values were obtained from the plateau phase of the binding curve obtained from CD spectroscopy measurements showing insignificant differences for either WT-αS or acetylated-αS indicating a similar size of helical domain on lipid membranes (*panel F*). All measurements were performed at room temperature in the presence of 10 mm Tris, 100 mm KCl buffered at pH 7.4. The *error bars* in all binding curves represent standard deviations from 3 independent measurements. The binding curves for LUVs (*open symbols*) shown in *panels C* and *E* could not be fitted using the solution to a simple quadratic equation (23) and the depicted lines are only a guide to the eye.

The strength of αS/lipid phospholipid membrane interactions is often quantified using spectroscopic methods, in particular CD spectroscopy (6, 26, 27), fluorescence correlation spectroscopy (28), and pulsed EPR (29, 30). Recent studies using NMR suggest that NTAc-αS has a slightly higher affinity than WT-αS for phospholipid vesicles (18, 31). To systematically characterize the binding of both acetylated-αS and WT-αS to phospholipid membranes in more detail using CD spectroscopy (26), we varied the percentage of the anionic lipid POPS (100, 75, 50, 25, and 0%) in POPC:POPS SUVs and LUVs. We characterized the size distribution and surface charge of lipid vesicles using dynamic light scattering and ζ potential measurements. Although the ζ potential varies as expected with increasing fraction of charged lipid used, the mean size of the different vesicles is essentially unchanged (∼60 nm for SUVs and ∼126 nm for LUVs). To be able to compare αS binding to the membranes of various phospholipid compositions, we determined the phospholipid concentration at which 50% of the αS was bound to vesicles (L_50_) and equilibrium dissociation constants (*K_d_*). Under our experimental conditions, the L_50_ approximates the *K_d_* values. The L_50_ values as a function of the fraction of anionic lipids are given in Table 1. The L_50_ values for both acetylated-αS molecules are comparable for all percentages of POPS in SUVs tested and show little difference (see also Fig. 2, *A–E, solid symbols* for binding curves) from the L_50_ values found for WT-αS with the exception of POPC SUVs (Fig. 2*E, solid symbols*). Binding of WT-αS to POPC SUVs was slightly weaker than binding of acetylated-αS. Considering that acetylated-αS has considerable α-helical structure in solution (18), the loss in conformational entropy upon binding to phospholipid membranes is probably lower for acetylated-αS than that for the unstructured WT-αS. Because the final helical content of both membrane-bound proteins is comparable (Fig. 2*F*), the net free energy gain upon binding of WT-αS to POPC membranes is larger, resulting in a slightly lower affinity of WT-αS for membranes of zwitterionic lipids. Upon increasing the fraction of POPS in the phospholipid membrane, electrostatic interactions between lysine residues and negatively charged headgroups dominate and likely mask the contribution of the conformational entropy.

**TABLE 1:** **L_50_ values (μm) of monomeric αS for different lipid compositions**

Lipid and protein	POPS (100)	POPC:POPS (25:75)	POPC:POPS (50:50)	POPC:Chol (50:50)	Chol:POPS (50:50)	POPC:POPS (75:25)	POPC (100)
**SUVs**							
**Liposome diameter (nm)**	65 ± 4	63 ± 3	60 ± 6	69 ± 8	67 ± 7	58 ± 2	64 ± 3
**Zeta potential, ζ (mV)**	−21.8 ± 1.3	−19.8 ± 0.8	−16.8 ± 1.5	−1.8 ± 0.8	−14.6 ± 2.5	−12.6 ± 0.9	−2.9 ± 0.6
**WT-αS**	57 ± 4	333 ± 7	294 ± 8	>2500*^a^*	539 ± 30	638 ± 11	2847 ± 137
**NTAc-αS**	54 ± 4	302 ± 10	310 ± 18	>2500*^a^*	455 ± 32	576 ± 15	1905 ± 65
**Endo-αS**	57 ± 5	257 ± 18	263 ± 16	>2500*^a^*	445 ± 31	524 ± 26	1967 ± 136
**LUVs**							
**Liposome diameter (nm)**	126 ± 4	ND*^b^*	126 ± 3	ND	ND	ND	128 ± 4
**Zeta potential, ζ (mV)**	−31.5 ± 1.2	ND	−23.1 ± 1.1	ND	ND	ND	−5.2 ± 0.3
**WT-αS**	572 ± 72	ND	>2000*^a^*	ND	ND	ND	>2500*^a^*
**NTAc-αS**	500 ± 32	ND	>2000*^a^*	ND	ND	ND	>2500*^a^*
**Endo-αS**	547 ± 21	ND	>2000*^a^*	ND	ND	ND	>2500*^a^*

*^a^* The binding data in these cases could not be fitted to the binding equation.

*^b^* ND, not determined.

Next, we investigated the influence of N-terminal acetylation on the curvature-dependent membrane binding of αS. It is known that WT-αS binds better to SUVs (30–60 nm diameter) than LUVs (100–200 nm diameter) (32). The higher affinity of WT-αS possibly results from the presence of intrinsic defects in SUVs, which result in increased exposure of the hydrophobic acyl regions to αS (26, 33). Table 1 shows that with decreasing liposome curvature the L_50_ values increase by at least an order of magnitude for the POPS liposomes as reported previously (32). As shown in Figs. 2, *C* and *E,* and 3*B* (*open symbols*), we could not determine the L_50_ values for the LUVs composed of 1:1 POPC:POPS or POPC or 1:1 POPC:Chol because hardly any phospholipid membrane binding was detectable by CD spectroscopy. Previous reports comparing the binding of NTAc-αS and WT-αS to SUVs and LUVs of similar equimolar mixtures of anionic and neutral phospholipids (DOPS and DOPC/DOPE) using NMR found no significant influence of acetylation on the apparent dissociation constants (31). Although we observe a lower affinity of αS to LUVs of most POPC:POPS mixtures compared with SUVs of the same composition, the L_50_ values for both acetylated-αS species are comparable with the values for WT-αS indicating that acetylation has no significant influence αS binding to liposomes. N-terminal acetylation only seems to affect the (weak) binding of αS to zwitterionic POPC vesicles.

**FIGURE 3.**
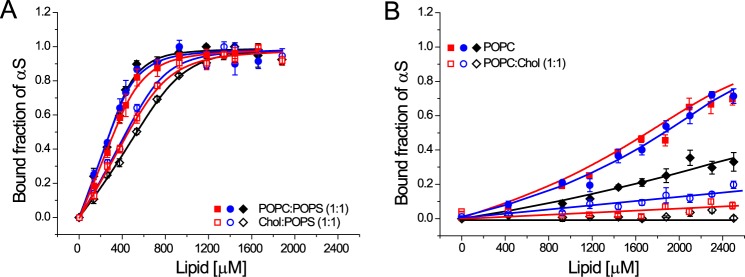
**Membrane binding characteristics of WT-αS, NTAc-αS, and Endo-αS to SUVs containing cholesterol.** *A,* binding curves comparing the affinity of αS to 1:1 Chol:POPS SUVs (*open symbols*) and 1:1 POPC:POPS SUVs (*closed symbols*). *B,* binding curves of αS to 1:1 POPC:Chol SUVs (*open symbols*) and POPC SUVs (*closed symbols*). The binding curves for SUVs (*open symbols*) shown in *panel B* could not be fitted using the solution to a simple quadratic equation (26) and the depicted lines are only a guide to the eye. All measurements were performed at room temperature in the presence of 10 mm Tris, 100 mm KCl buffered at pH 7.4. The *error bars* in all binding curves represent standard deviations from 3 independent measurements.

Cholesterol is a critical component of cellular membranes and has been shown to affect the binding of αS (34). Estimations of the plasma membrane composition in existing literature report an equimolar ratio of cholesterol and phospholipids (35, 36). To test the effect of the presence of cholesterol on the binding of acetylated-αS, we used vesicles composed of 1:1 mixtures of cholesterol and either POPC or POPS. We observed that the presence of cholesterol in SUVs of the anionic lipid POPS decreases the binding affinity by ∼2-fold for acetylated-αS and WT-αS (Fig. 3*A*). Inclusion of cholesterol in SUVs of the zwitterionic phospholipid POPC nearly abolished membrane binding of acetylated-αS and WT-αS (Fig. 3*B*) in CD spectroscopy measurements. Given the comparable values of ζ potentials for POPC and POPC:Chol (1:1) SUVs (Table 1), the apparent observation of abolished membrane binding to POPC SUVs upon incorporation of cholesterol cannot be explained as a result of change in surface charge of the lipid vesicles. It is known that cholesterol can promote the lipid ordering at the equimolar phospholipid/cholesterol ratios used in our study (37–39) and the reduced affinity of αS for such ordered lipid phases (40) may explain the decreased binding of αS to membranes used in our study. N-terminal acetylation although, does not seem to have any significant effect on binding of αS to cholesterol containing model membranes.

**FIGURE 4.**
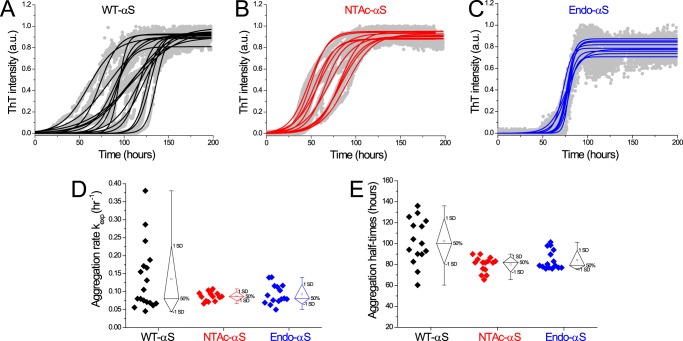
**Aggregation kinetics of WT-αS, NTAc-αS, and Endo-αS at 37 °C monitored by measuring ThT fluorescence.** The aggregation reaction was carried out with a protein concentration of 35 μm WT-αS (*black symbols*), NTAc-αS (*red symbols*), and Endo-αS (*blue symbols*) using PBS buffer at 300 rpm in a TECAN fluorescence microplate reader at 37 °C (*panels A–C*). The exponential phase aggregation rates (*panel D*) and the corresponding aggregation half-times (*panel E*) were obtained from the aggregation curves as mentioned elsewhere (10). The ThT concentration was 5 μm.

**FIGURE 5.**
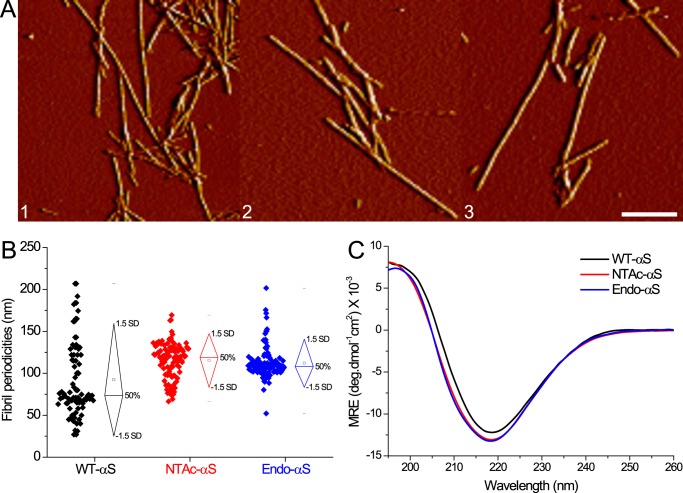
**AFM and CD spectroscopy of WT-αS and acetylated-αS fibrils.** *A,* AFM amplitude images depicting fibrillar aggregates of WT-αS (*panel 1*), NTAc-αS (*panel 2*), and Endo-αS (*panel 3*). The *scale bar* is 250 nm. *B,* fibril periodicities measured from AFM images show slightly higher values for acetylated-αS compared with WT-αS. *C*, CD spectroscopy of purified αS fibrils show slightly higher β-sheet content in acetylated-αS fibrils than WT-αS fibrils. All fibrils were prepared in PBS buffer solutions and purified after aggregation to remove monomers.

**FIGURE 6.**
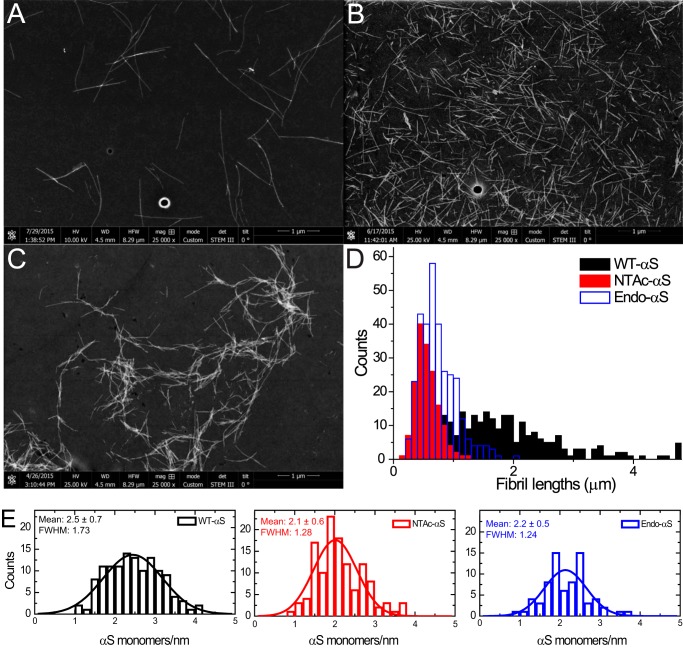
**STEM measurements of WT-αS, NTAc-αS, and Endo-αS fibrils.** Representative dark-field images of WT-αS (*panel A*), Endo-αS (*panel B*), and NTAc-αS (*panel C*) fibrils obtained post-aggregation in PBS buffer at 37 °C with constant shaking at 300 rpm. The fibrils were purified with a 100-kDa cutoff filter to remove the residual monomers before STEM imaging. The length distributions (*panel D*) were obtained using the *Simple Neurite Tracer* plugin (69) in Fiji software show a much smaller mean length acetylated-αS fibrils as compared with WT-αS fibrils. Histograms of mass per length measurements and their corresponding fitted Gaussian distributions are depicted as *solid curves* (*panel E*). Statistical analysis using one-way analysis of variance predict that the sample mean of WT-αS is significantly different from both acetylated-αS at *p* < 0.05.

Although we did not observe significant changes in phospholipid membrane binding affinity of αS after acetylation, the acetylation may affect the tendency of αS to aggregate into amyloid fibrils. Impact of N-terminal αS acetylation on its aggregation rate is unclear, with contradicting reports in the existing literature (18–20, 41). To probe the influence of acetylation on aggregation into amyloid fibrils, fibril growth was examined using a thioflavin T (ThT) fluorescence assay. The normalized ThT fluorescence of acetylated-αS and WT-αS exhibit a typical sigmoidal shape (Fig. 4, *A–C*). The aggregation lag times and aggregation rates obtained from sigmoidal fits are highly variable for WT-αS, whereas narrower distributions are found for the acetylated-αS (Fig. 4, *D* and *E*). Interestingly, this smaller variability in the lag times observed in ThT-aggregation curves for N-terminal acetylated-αS was observed earlier (42) but the authors did not elaborate on this observation. Although surface induced aggregation (5) can lead to variability in fibrillization kinetics, both WT-αS and acetylated-αS monomeric samples were monitored on the same microplate under identical conditions. It is therefore reasonable to assume that the heterogeneity in fibrillization kinetics reported by ThT is a result of N-terminal acetylation in αS. The narrow lag time distribution observed for acetylated-αS compared with WT-αS suggests that acetylation results in the nucleation of a more homogenous population of fibrils. Morphological analysis of samples obtained at the plateau phase of ThT fluorescence using AFM and scanning transmission electron microscopy (STEM) confirmed that both acetylated-αS and WT-αS formed fibrillar aggregates (Fig. 5*A*). Fibril heights of WT-αS and both acetylated-αS species obtained from AFM images are comparable, whereas the fibril periodicity (helical pitch of the twisted fibrils) distributions indicate that acetylated-αS fibrils have slightly higher periodicities (Table 2). The periodicity distribution of WT-αS fibrils is much broader compared with that of acetylated-αS fibrils (Fig. 5*B*). The spread in the periodicity distribution possibly reflects the heterogeneity in aggregation rates observed in ThT experiments. The presence of EDTA in aggregation mixtures has been reported to result in homogenous fibril preparations possibly by restriction of conformations accessible to a monomer (43). The mean fibril length of WT-αS was ∼3-fold higher than that of acetylated-αS fibrils (Fig. 6, *A–D*). Because fibril lengths can be influenced by stochastic shear forces arising during sample preparation, it cannot be ascertained conclusively if differences in the apparent mean fibril lengths result from acetylation of αS. Dark-field STEM images of filamentous structures can be readily quantified to obtain the mass per length (MPL); a concept commonly known as mass mapping (44). Using tobacco mosaic virus (TMV) rods as a calibration standard, we obtained molecular level information on both acetylated-αS and WT-αS fibrils (Fig. 6*E*). Assuming one main population of fibrillar species, the mean mass per unit length was obtained by fitting a single Gaussian to the obtained distribution. For WT-αS fibrils, a mean MPL of ∼75 kDa/nm was obtained, whereas we observed a mean MPL of ∼66 kDa/nm for both acetylated-αS fibrils (equivalent to ∼2.5 and ∼2.1 subunits/nm, respectively). The full width at half-maximum (FWHM) values for WT-αS fibrils are higher compared with acetylated-αS fibrillar structures. The observation that the acetylated-αS fibril population is structurally more homogenous is in agreement with the narrow periodicity and lag time distribution observed from AFM measurements. The mean MPL value of ∼75 kDa/nm (∼2.5 subunits/cross-section) obtained for WT-αS fibrils is slightly higher than the recently reported value of ∼59 kDa/nm (∼1.9 subunits/cross-section). This difference possibly results from the higher ionic strength (137 mm NaCl) used here compared with the previous study (100 mm NaCl).

**TABLE 2:** **Overview of structural parameters for αS fibrils obtained from atomic force microscopy (AFM) and scanning transmission electron microscopy (STEM)** Fibril heights (nm) and periodicities (nm) were measured from AFM images and mean fibril lengths (μm) from STEM images. Error bars represent standard deviations.

	Fibril height	Fibril periodicity	No. of fibrils (AFM)	Mean fibril length	No. of fibrils (STEM)
	*nm*	*nm*	*n*	μ*m*	*N*
**WT-αS**	6.8 ± 1	84 ± 44	83	1.83 ± 0.8	124
**NTAc-αS**	6.1 ± 1	115 ± 12	108	0.54 ± 0.2	245
**Endo-αS**	6.7 ± 1	112 ± 19	113	0.70 ± 0.3	194

Acetylation seems to also influence the conformational ensemble of the monomeric αS in solution as evidenced by NMR measurements (18) and may thereby also affect the nucleation of a more homogenous population of fibrils. The differences in fibril morphology are also reflected in the secondary structure observed for αS fibrils. Although the CD spectroscopy showed a characteristic negative peak at ∼218 nm for both acetylated-αS and WT-αS fibrils, the acetylated-αS fibrils had slightly higher β-sheet content (Fig. 5*C*). Similar differences in calculated CD spectra have been recently reported for αS with and without N-terminal acetylation by molecular dynamics (MD) simulations (45). The broader fibril periodicity distribution observed for WT-αS fibrils and the differences between the CD spectra of WT-αS and acetylated-αS fibrils may result from a difference in molecular conformation.

**FIGURE 7.**
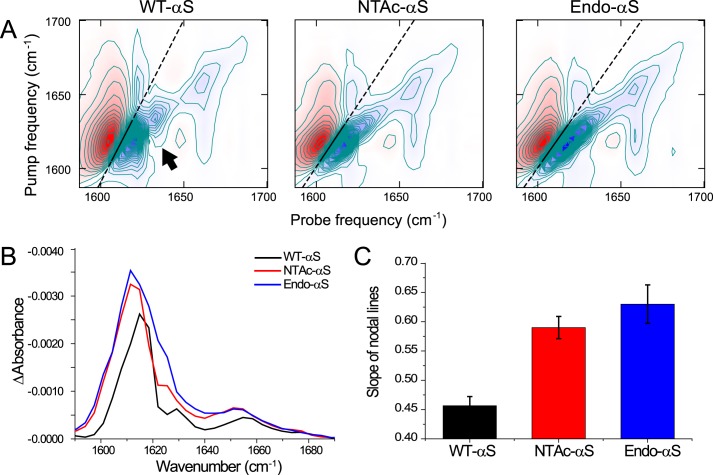
**2D-IR spectra of αS fibrils.** *A,* 2D-IR spectra showing *solid straight lines* that are fits through the zero crossings in the β-sheet region. The steeper slope of the line in the WT-αS fibril spectrum shows that the spectral heterogeneity is less in this spectrum as compared with the acetylated-αS spectra. The *arrow* indicates a cross-peak between the ∼1620 cm^−1^ and 1632 cm^−1^ peaks, which is only present in the WT-αS spectrum, indicating coupling between modes resulting from two different types of β-sheet structure. All fibrils were prepared in deuterated PBS buffer solutions and purified after aggregation to remove monomers. *B,* diagonal slices of the 2D-IR spectra to aid the recognition of the diagonal peaks described in the main text. To avoid distortion of the line shapes as a result of a large spectral width of the pump as compared with the anharmonicity that results in a distorting positive contribution of the induced absorption to the bleach signal that is plotted here, we plot the average between the diagonals that are blue shifted by one and two probe pixels. *C,* the nodal slopes that were obtained from the fitted straight lines through the zero crossings in the β-sheet region, showing a comparable spectral inhomogeneity for acetylated-αS fibrils, and a smaller inhomogeneity for WT-αS fibrils. We obtained the nodal slopes by calculating the frequencies where the signal goes through zero, between the induced absorption (*red peak* at lower probe frequency in *panel A*) and the bleach (*blue peak* at higher probe frequency in *panel A*), for each pump pixel in the 1600–1622 cm^−1^ region by interpolation of the data point right before and right after the zero crossing, and subsequently fitting a straight line through the interpolated zero crossings.

To investigate this possibility, we measured 2D-IR spectra in the amide-I region (1600–1700 cm^−1^), which provide information on secondary protein structure (46–50). There are significant differences between the 2D-IR spectra of WT-αS fibrils and acetylated-αS fibrils (Fig. 7, *A* and *B*). We assign the four IR-active modes (visible on the diagonal of the 2D-IR) spectra as follows: the peak at (ν_probe_, ν_pump_) = (1657, 1657) cm^−1^ is indicative of turns (51, 52), and the peaks at (1620, 1620) cm^−1^, (1632, 1632) cm^−1^, and (1683, 1683) cm^−1^ are indicative of β-sheet structure (52–55) (Fig. 7*C*).

**FIGURE 8.**
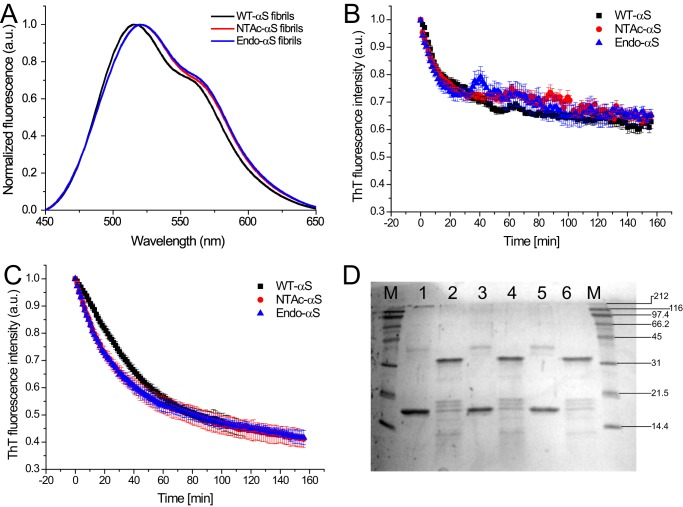
*A,* fluorescence emission spectra of FE-dye bound to αS fibrils. 20 μm WT-αS (*black*), NTAc-αS (*red*), and Endo-αS fibrils (*blue*) were incubated for 1 h with 2 μm FE-dye in PBS buffer at room temperature. The fluorescence emission spectra were acquired using an excitation wavelength of 420 nm and excitation/emission slit widths at 5 nm. *B,* stability of αS fibrils to urea exposure followed by ThT fluorescence. Comparable fibril denaturation rates and loss of β-sheet content were observed for WT-αS and the acetylated protein fibrils. *C,* proteinase K digestion assay wherein the β-sheet content of the fibril solution was followed by ThT fluorescence. The data points in *panels B* and *C* represent mean ± S.D. of a minimum 3 independent measurements. *D,* the corresponding Coomassie-stained SDS-PAGE (12%) gel. Standard molecular weight markers (*lane M*) are shown on the *right side* of the gel. Undigested αS fibrils were loaded in *lanes 1* (WT-αS), *3* (NTAc-αS), and *5* (Endo-αS), whereas proteinase K-digested fibrils after completion of experiment were loaded in *lanes 2* (WT-αS), *4* (NTAc-αS), and *6* (Endo-αS).

The most notable spectral differences distinguishing WT-αS from NTAc-αS fibrils are the cross-peak patterns and the spectral inhomogeneity. The cross-peak at (ν_probe,_ ν_pump_) = (1657, 1620) cm^−1^ shows that the vibrational modes in the turns are spatially close enough to couple to the vibrational modes in the β-sheets. Likewise, the cross-peak in the WT-αS spectrum at (1632, 1620) cm^−1^ (*arrow* in Fig. 7*A*) reveals vibrational coupling between different β-sheet modes. The latter cross-peak is not observed in the spectra of the acetylated-αS, indicating a clear structural difference. The slanted shape of the diagonal peaks indicates spectral inhomogeneity: when scanning the excitation frequency ν_pump_ over the absorption band, the response shifts to higher ν_probe_ with increasing ν_pump_ (in the absence of spectral inhomogeneity the peak shape is parallel to the ν_pump_ axis) (46). If there are many oscillators with a slightly different environment leading to a large spectral inhomogeneity, the slope of the response will go toward 45°. WT-αS fibrils have a relatively smaller degree of spectral heterogeneity in the β-sheet region than acetylated-αS fibrils, which is evinced by the different slopes of the nodal lines (46) (*black lines* in Fig. 7*A* and their corresponding slopes in Fig. 7*C*). This increased spectral heterogeneity of acetylated-αS fibrils can be due to increased solvent exposure of the β-sheets, and/or to a broader conformational distribution (46, 56). The former scenario is not likely, because experiments using the polarity-sensitive FE-dye (57) show that the core of acetylated-αS fibrils is just as polar as that of WT-αS fibrils (Fig. 8*A*).

To further characterize the structural properties of WT-αS and acetylated-αS fibrils, we tested their stability in 4 m urea (Fig. 8*B*) and susceptibility to proteinase K digestion (Fig. 8*C*) by monitoring the loss in β-sheet content in ThT assays as a function of time. Both WT-αS and acetylated-αS fibrils show similar susceptibilities to 4 m urea after ∼3 h, whereas WT-αS fibrils seemed to be slightly more resistant to proteolytic cleavage than acetylated-αS fibrils. However, this difference is not significant as the band pattern observed in SDS-PAGE (Fig. 8*D*) shows an identical number of bands for both WT-αS and acetylated-αS fibrils. This observation indicates that the same proteolytic cleavage sites are exposed in fibrils of WT and acetylated-αS. The smaller fibril-to-fibril heterogeneity of acetylated-αS as compared with WT-αS fibrils as measured by AFM suggests that the larger spectral inhomogeneity observed in the 2D-IR measurements for the acetylated-αS fibrils is not the result of a random distribution of structures, but of a well defined distribution of different β-sheet structures present within one fibrillar repeating unit (58). Recent microelectron diffraction experiments indicated insignificant differences in the intermolecular spacing of β-sheets of NTAc-αS and WT-αS, which also explains similarities in heights of NTAc-αS and WT-αS fibrils from our AFM experiments (59). This conclusion is also supported by similar fibril denaturation susceptibilities of WT-αS and acetylated-αS fibrils to degradation by 4 m urea and proteinase K. The similarities in fibril structures and vibrational signatures of acetylated-αS fibrils in our measurements thus suggest that NTAc-αS faithfully mimics Endo-αS, the purification of which is cumbersome.

Under our experimental conditions, N-terminal acetylation seems to have little influence on membrane binding of αS to phospholipid membranes. In line with this observation the subcellular localization and distribution of αS has been observed to be unaffected by N-terminal acetylation (20). This suggests that if N-terminal acetylation of αS plays a regulatory role in the function of the protein, it should act in conjunction with either a physicochemical cue or another binding partner. N-terminal acetylation in αS may not be directly used to tune membrane binding but is possibly required to adjust the interaction strength with other partners like soluble *N*-ethylmaleimide-sensitive factor attachment receptors (SNAREs), actin (1), tubulin (60, 61), or specific lipids (41). Further studies targeted at elucidating binding partners of monomeric αS could yield more insight into the impact of N-terminal acetylation in regulating interactions. Although we do not observe major differences in aggregation rates of both acetylated-αS and WT-αS, N-terminal acetylation does result in a high degree of homogeneity in aggregation lag times and fibril morphologies (Table 3). Structural polymorphs of αS and Aβ fibrils have been shown to result in significantly different toxicities in neuronal cell cultures (62–64) and considering that *in vitro* preparations of WT-αS fibrils have significant polymorphism, acetylated-αS fibrils are more relevant for such studies.

**TABLE 3:** **Effect of N-terminal acetylation on biophysical properties of αS**

	Probed parameter	Technique used	Effect of N-terminal acetylation
**Primary structure**	Subcellular localization/distribution	Fluorescence microscopy	No significant effect (17. 20)
	Primary native structure	Mass spectrometry, SDS-PAGE, Native-PAGE, CD spectroscopy	Monomeric (15, 18, 20 and this paper) Tetrameric (16. 72)
**Secondary structure**	Membrane binding of αS monomer	CD spectroscopy, Isothermal calorimetry, nuclear magnetic resonance	Enhanced binding to GM1 gangliosides (41) Comparable binding to GM3, POPS lipids (18, 31, 41)
**Aggregation properties**	Amyloid formation rate	Thioflavin T fluorescence	Two-fold decrease (19. 73)
			No significant effect (18. 20)
	Aggregation lag-time variability	Thioflavin T fluorescence	Decreases (19 and this paper)
**Fibrillar structure**	Fibril height(nm)	Atomic force microscopy	No significant effect (this paper)
	Secondary structure	CD spectroscopy of fibrils	Increased β-sheet content (45 and this paper)
	High resolution Secondary structure	2D-IR spectroscopy	Increased fibril homogeneity (this paper)
	Solvent exposure of fibril core	Fluorescence spectroscopy	No significant effect (this paper)
	Urea digestion assay	Thioflavin T fluorescence	No significant effect (this paper)
	Proteinase-K digestion assay	Thioflavin T fluorescence and SDS-PAGE	No significant effect (this paper)
	Mass mapping	Scanning Transmission Electron Microscopy (STEM)	- 2 monomers per nm (71) - 2–3 monomers per nm of fibril (this paper)

## Experimental Procedures

### 

#### 

##### Expression, Purification, and Labeling of αS

WT-αS was expressed in *Escherichia coli* strain BL21(DE3) using the pT7-7 expression plasmid and purified in the presence of 1 mm DTT as previously reported (65). Endogenous αS was purified from freshly collected human RBCs provided by Sanquin blood bank, The Netherlands. The purification protocol used is similar to that described elsewhere (16), except using first an anion exchange column for bulk purification (GE Healthcare, Source 15Q) followed by further purification with a hydrophobic interaction column (GE Healthcare, HiTrap Phenyl HP). NTAc-αS protein was produced by co-expression of both the αS plasmid and the N-terminal acetylation B complex plasmid in *E. coli*. The *N*-acetylation B complex plasmid was kindly provided by Dr. Daniel Mulvihill. The purification protocol is the same as for WT-αS. All protein samples were confirmed to be monomeric from acetic acid gel electrophoresis.

##### Mass Spectrometry

Electrospray ionization (ESI) mass spectra were acquired on a Thermo Finnigan LTQ FT-ICR in positive mode. The sample was inserted by means of a syringe pump. The spray voltage was operated between 1 and 1.5 kV. The final concentration of αS monomers was 15 μm in 10 mm ammonium acetate buffer.

##### Acetic Acid Gel Electrophoresis

Proteins were separated based on the difference in acetylation of the N terminus by acetic acid-urea polyacrylamide gel electrophoresis using a protocol as described elsewhere (66).

##### Preparation of Liposomes

Stock solutions of 1-palmitoyl-2-oleoyl-*sn*-glycero-3-phosphocholine (POPC), 1-palmitoyl-2-oleoyl-*sn*-glycero-3-phospho-l-serine (POPS), and cholesterol (Chol) from ovine wool were purchased from Avanti Polar Lipids (Birmingham, AL) and used without further purification. Tris salt and potassium chloride (KCl) were purchased from Merck (Germany). Lipid stock solutions of POPC and POPS in chloroform were mixed in appropriate molar ratios, dried under a stream of nitrogen, and placed under vacuum for 1 h. After drying the lipid films were rehydrated in 10 mm Tris, 100 mm KCl solution and vortexed for 5 min. SUVs were prepared by sonicating the rehydrated liposome solution for 40 min using a Branson tip sonicator. Thereafter, the SUVs were centrifuged at 16,100 × *g* to remove any tip residue from the sonicator probe. For preparation of LUVs, the rehydrated liposome solution (after the vortexing step) was subjected to multiple cycles of freeze-thawing in liquid nitrogen until the resulting solution was clear. Thereafter, the solution was extruded through a polycarbonate membrane of pore size 100 nm. The SUVs and LUVs were used immediately after preparation.

##### Dynamic Light Scattering and ζ Potential Measurements

The size and ζ potential of the lipid vesicle solutions were characterized on a Malvern Zetasizer Nano ZS (Malvern Instruments, UK). For dynamic light scattering measurements, lipid vesicles in 10 mm Tris, 100 mm KCl were prepared and 10 acquisitions were performed for each sample at room temperature. For the ζ potential measurements, lipid vesicles were added to capillary cells with integral gold electrodes. The values of ζ potential were obtained directly from the Zetasizer software using the Smoluchowski approximation. More than five measurements, each consisting of 30 runs, were performed for every sample at room temperature. The ζ potentials and vesicle diameters of the different vesicles used in the study are listed in Table 1.

##### CD Spectroscopy

A Jasco J-1500 spectropolarimeter was used to obtain CD spectra at a protein concentration of 3 μm in phosphate-buffered saline (PBS) containing 10 mm phosphate buffer, 137 mm NaCl, 2.7 mm KCl, pH 7.4. By measuring the increase in absorbance at 222 nm that is indicative of a transition of the monomeric protein from a random to a helical conformation upon lipid association, a binding curve could be generated by titrating αS with liposomes. The binding curves were then normalized assuming saturation of mean residual ellipticities (MRE) values in the plateau phase of the binding curve represents saturation of protein binding sites on the lipid membrane. The normalization of the binding curve for incomplete saturation (in case of zwitterionic membranes) was performed using average MRE values obtained at saturation conditions for the respective variant of αS. Fitting of the binding curves was done using a binding equation as reported before (26). Aggregation of monomeric αS was carried out in PBS buffer at 37 °C under constant orbital shaking at 300 rpm. For measurement of CD spectra of αS fibrils, fibril samples were first purified using a 100-kDa cut-off filter to remove monomeric αS. Thereafter, CD spectra were recorded between 195 and 260 nm with a step size of 1 nm and a scanning speed of 10 nm/min using a 1-mm path length cuvette at room temperature.

##### ThT Aggregation Assay

All aggregation assays were carried out in a TECAN InfinitePro200 multiplate fluorescence plate reader on standard polystyrene microplates using a protein concentration of 35 μm in PBS buffer at 37 °C under constant orbital shaking at 300 rpm. The ThT concentration was 5 μm. Protein samples were purified using a 100-kDa cut-off filter prior to beginning of measurements to ensure that no aggregates were present. The exponential phase aggregation rates and the corresponding aggregation half-times were obtained from the aggregation curves as mentioned elsewhere (10).

##### Atomic Force Microscopy

For AFM measurements, 20 μl of 10 μm fibril suspension was incubated on freshly cleaved mica (15 × 15 mm) for 5 min at room temperature. Samples were thereafter washed with MilliQ water and dried using N_2_ gas. AFM images were acquired in tapping mode on a Dimension 3100 Scanning Probe Microscope (Bruker) using NSG01 gold probes with a resonant frequency between 87 and 230 kHz and a tip radius ∼10 nm. For preparation of fibrils, identical aggregation conditions were employed: 35 μm monomeric αS in PBS buffer at 37 °C under constant orbital shaking at 300 rpm. Fibril heights were measured using NanoScope Analysis version 1.5 software and for the measurements of periodicities (helical pitch of the twisted fibrils), fibrils in AFM images were traced using a custom written script in MATLAB using the DIPimage toolbox (version 2.3, TU Delft, Delft, The Netherlands) was used (67). The script is based on quantitative analysis of AFM images mentioned elsewhere (65).

##### 2D-IR Spectroscopy

The 2D-IR spectra were measured on a setup described elsewhere (68). In short, a commercially available mode-locked Ti:sapphire oscillator system whose output is amplified by a Ti:sapphire regenerative amplifier was used to create 35 fs, 800-nm pulses of ∼3.1 mJ at a repetition rate of 1 kHz. These were converted in an optical parametric amplifier into ∼100 fs, ∼6100 nm pulses of ∼20 μJ with an approximately Gaussian distribution that has a FWHM of ∼150 cm^−1^. The IR beam was then split into a pump, probe, and a reference beam. The pump beam is led through a Fabri-Perrot interferometer, and thereby reduced in bandwidth to a FWHM of ∼12 cm^−1^. The pump beam was then rotated 90° with respect to the probe beam by a λ/2 plate, and subsequently overlapped with the probe pulse in the sample in a ∼200 μm focus. All spectra were obtained at a pump-probe delay of 1.5 ps. After the sample, the probe and reference beam were coupled into an OrielMS260i spectrograph that disperses the light onto a 32 pixel MCT-array with a resolution of 3.9 cm^−1^. Fibril samples for 2D-IR measurements were prepared in deuterated PBS buffers at 37 °C, 300 rpm constant shaking in Eppendorf® LoBind tubes. Prior to measurements, monomers were removed via centrifugation at 10,290 × *g*.

##### Scanning Transmission Electron Microscopy

For preparation of fibrils, monomeric αS samples were aggregated in PBS buffer at 37 °C under constant orbital shaking at 300 rpm, diluted with MilliQ water, and then prepared for STEM dark-field imaging. Typically, a 5-μl drop of 20 μm fibril samples were adsorbed on 300 mesh formvar-coated copper grids for 5 min and then washed 5 times with water. The grids were thereafter dried at 37 °C and then transferred under vacuum into the STEM setup. Dark-field digital images of fibrils were acquired using a FEI Verios 460 microscope operating at 25 kV electron beam energy using the high-angle annular dark-field detectors. Before recording the dark-field STEM images, condenser stigmators were carefully adjusted to give a circular beam profile when the beam was viewed on the grids, and the beam was carefully centered and spread to produce uniform illumination over the field of view. Histograms for fibril length were obtained from these data using the *Simple Neurite Tracer* plugin in Fiji software (69, 70). For mass mapping measurements, a 2-μl drop of TMV rods (100 μg/ml stock in 10 mm Tris buffer) was adsorbed for 2 min to imaging grids washed several times with MilliQ water. Thereafter, a 5-μl drop of 20 μm fibril samples were allowed to adsorb for 5 min followed by drying at 37 °C. Images were analyzed using ImageJ software following the protocol mentioned elsewhere (44). Measurements were converted from mass to subunits using a subunit mass of 14.46 kDa for WT-αS and 14.50 kDa for acetylated-αS. The Gaussian mean is shown in the respective panels along with the FWHM kDa/nm; suggesting ∼2–3 αS subunits per 0.47 nm (*n* ≧ 100).

##### Fibril Denaturation Assay

αS (both acetylated and non-acetylated) fibrils (0.2 mg/ml) in PBS buffer were treated at 37 °C with proteinase K (0.025 mg/ml). Immediately after proteinase K addition each sample was divided into two aliquots. ThT was added to a final concentration of 1 μm in the first aliquot and used to monitor changes in ThT emission fluorescence for ∼3 h. The second aliquot was incubated under identical conditions without addition of ThT and after ∼3 h, the samples were transferred to Eppendorf tubes maintained at 90 °C containing the running buffer to arrest immediately the cleavage reaction. After incubation of each tube for 5 min at 90 °C, the samples were loaded in a SDS-PAGE (12%) gel and stained later with Coomassie Blue.

## Author Contributions

A. I., M. M. A. E. C., and V. S. conceived the experiments. A. I., S. R., N. S., and B. H. performed the experiments. All authors, including S. W. and R. M. A. H., analyzed the results and contributed to writing of the paper. All authors approved the final version of the manuscript.

## Footnotes

5 The abbreviations used are:

αS: α-synuclein
POPC: 1-palmitoyl-2-oleoyl-*sn*-glycero-3-phosphocholine
POPS: 1-palmitoyl-2-oleoyl-*sn*-glycero-3-phospho-l-serine
NTAc: N-terminal acetylation
2D-IR: two-dimensional infrared spectroscopy
STEM: scanning transmission electron microscopy
SUVs: small unilamellar vesicles
LUVs: large unilamellar vesicles
ThT: thioflavin T
AFM: atomic force microscopy
Chol: cholesterol
MRE: mean residual ellipticities
PTM: post-translational modification
TMV: tobacco mosaic virus
FWHM: full width at half-maximum
MPL: mass per length.
